# Pulmonary vein stenosis or occlusion resulting from radiofrequency ablation of atrial fibrillation can be mistakenly diagnosed as pneumonia: a case report

**DOI:** 10.3389/fcvm.2024.1509227

**Published:** 2025-01-23

**Authors:** Jun xian Gong, Ai fen Zong, Yue zhen Gong

**Affiliations:** Department of Cardiology, Huantai County People’s Hospital, Zibo City, Shandong Province, China

**Keywords:** case report, atrial fibrillation, radiofrequency ablation, pulmonary vein stenosis or occlusion, pneumonia

## Abstract

The increased use of radiofrequency ablation (RFA) for atrial fibrillation (AF) has led to a rise in cases of pulmonary vein stenosis or occlusion (PVS/O) as a complication. While this occurrence was once rare, the growing number of patients undergoing this procedure has made it more prevalent. The actual frequency of pulmonary vein (PV) occlusion remains a topic of debate. This complication can present with non-specific respiratory symptoms, potentially leading to misdiagnosis and delayed treatment, with serious consequences. Clinical signs of PVS/O post-ablation can vary from no symptoms to common respiratory issues like coughing, hemoptysis, shortness of breath, and chest pain. Failure to promptly diagnose and address this condition may result in the need for lung lobectomy and even pose life-threatening risks.

## Patient information

**Figure 1:**
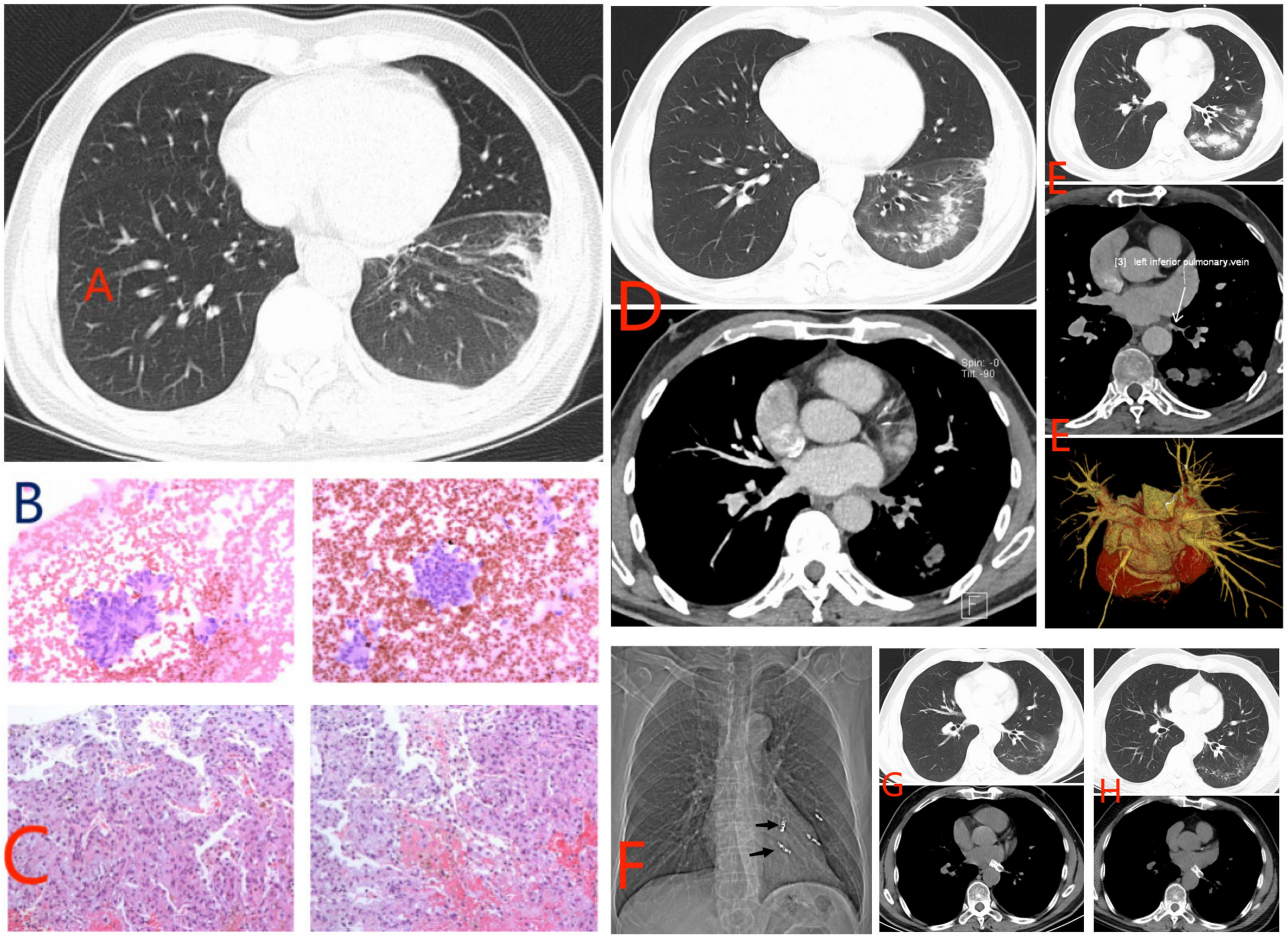
Information on ancillary tests for patients in clinical care. **(A)** Inflammation of the lower lobe of the left lung and pleural effusion. **(B)** Cytological examination. **(C)** Percutaneous lung biopsy. **(D)** Pulmonary artery CT scan. **(E)** Pulmonary vein contrast-enhanced CT scan shows left lower pulmonary vein occlusion. **(F)** Bleeding artery embolization. **(G)** Lung CT shows improvement in condition. **(H)** Review lung CT on March 1, 2024.

The case report describes a 53-year-old male patient who presented with paroxysmal palpitation in January 2018, leading to a diagnosis of paroxysmal AF. The patient had a history of hypertension but no other significant comorbidities. Coronary angiography showed mild stenosis of the anterior descending artery and right coronary artery, but no significant stenosis of the circumflex artery. Cardiac echocolor Doppler showed no abnormalities in cardiac function and pulmonary artery pressure. Despite occasional episodes of AF, the patient responded well to oral metoprolol succinate. Following a recurrence of AF in June 2021, the patient underwent RFA. No PV CT examination was performed before RFA. During the ablation of the PV vestibule, the power control mode was utilized at 40 watts, 43°C, and a flow rate of 30 ml/min. After achieving complete isolation of the pulmonary veins, voltage matrix mapping ablation was conducted using the power control mode at 35 watts, 43°C, and a flow rate of 25 ml/min. The patient subsequently received post-operative treatment with rivaroxaban and amiodarone. Subsequently, in January 2022, the patient experienced chest pain, coughing, chest tightness, and shortness of breath, with chest CT revealing inflammation in the lower lobe of the left lung and pleural effusion (Figure 1A). The patient was diagnosed with pneumonia and pleurisy, leading to admission to the Department of Respiratory Medicine where anti-infective treatment was administered. Following improvement, the patient was discharged. However, 1 month later, the patient experienced a recurrence of symptoms including cough, white sputum, occasional bright red sputum, persistent left chest and back pain, chest tightness, and shortness of breath after physical activity. Despite ruling out tuberculosis and connective tissue diseases through various examinations, sputum bacterial culture did not reveal any abnormalities. Cytological examination post bronchoscopy and alveolar lavage indicated the presence of red blood cells, respiratory epithelial cells, neutrophils, and lymphocytes, with no cancer cells detected (Figure 1B). A percutaneous lung puncture biopsy on April 21, 2022 revealed chronic non-specific interstitial inflammatory changes, edema, and scattered infiltration of lymphocytes, histiocytes, and neutrophils, with no granulomatous lesions or atypical cells observed. Tissue changes in the left lower lobe aspirate were consistent with pulmonary hemorrhage and infarction (Figure 1C). A pulmonary artery CT scan on April 22, 2022 confirmed left lower PV occlusion, reduced perfusion, and volume reduction in the left lower lobe of the lung (Figure 1D). The etiology of the patient's recurrent pneumonia was identified as PV occlusion following RFA for AF. Symptoms including cough, sputum production, shortness of breath, chest pain, and hemoptysis gradually manifested. Despite initial treatment with anti-infectious and anticoagulant therapies during the first hospitalization, persistent symptoms of cough and sputum production prompted a PV enhanced CT scan on July 26, 2022. The scan revealed occlusion between the left lower PV and the left atrium, with absence of contrast agent filling and presence of multiple patchy high-density shadows in the corresponding lung fields (Figure 1E). Subsequent interventions included arterial embolization to address recurrent hemoptysis resulting from pulmonary infarction (Figure 1F). Following this, the patient underwent the implantation of a stent in the left lower PV, measuring 9 mm in diameter and 20 mm in length. Postoperative management involved a combination of aspirin and clopidogrel for 3 months, followed by maintenance therapy with daily oral aspirin at 100 mg. The patient showed gradual improvement in symptoms such as cough, sputum production, and shortness of breath. A follow-up chest CT on March 30, 2023 revealed a dense shadow of the stent in the left lower PV and decreased density of embolic lesions in the left lung, with no mediastinal lymphadenopathy or pleural effusion detected. The patient experienced gradual relief of symptoms and a chest CT scan confirmed a significant reduction in the size of lesions in the lower lobe of the left lung (Figure 1G). Following the placement of a left lower PV stent, the patient's clinical symptoms improved markedly. The patient underwent a review of chest CT on March 1, 2024 (Figure 1H). From the CT images, his condition improved significantly. Cardiac echocolor Doppler examination showed pulmonary artery pressures within normal limits. The patient's current condition remains stable, with no significant restenosis observed within the PV stent. It is important to note that the initial symptoms of PVS/O can be non-specific respiratory symptoms, leading patients to seek treatment in respiratory medicine departments. However, there may be a lack of awareness among respiratory medicine physicians regarding this complication, potentially resulting in misdiagnosis and delayed treatment. The timeline of the patient's journey through clinical care can be found in Table 1.

**Table 1:** Important time points and therapeutic measures in the clinical care of patients.

Time	Event
2018-01	Atrial fibrillation occurred for the first time
2021-06-18	Radiofrequency ablation of atrial fibrillation
2022-01	The patient developed left chest and back pain, accompanied by cough and chest tightness after activity.
2022-04-21	Percutaneous lung biopsy
2022-04-22	Pulmonary artery CT
2022-07-26	Pulmonary vein CT
2022-09	Bleeding artery coil closure and left lower pulmonary vein stent implantation were performed in September 2022.
2023-03	Review lung CT
2024-03-01	The patient's lung CT review

## Discussion

PVS/O is a rare condition with potentially serious consequences if not promptly diagnosed and treated. PVS may arise from congenital, acquired, or iatrogenic sources. The primary cause of acquired PVS/O is RFA of AF, which isolates the pulmonary veins electrically and creates conduction block. Excessive thermal damage during this procedure can lead to fibrosis, scarring, and intimal proliferation, ultimately resulting in PVS/O. The increasing use of pulmonary vein isolation (PVI) for AF has led to another PVS cohort. Although the incidence of severe AF ablation-induced PVS/O in experienced cardiac electrophysiology centers is less than 1%, the increasing number of AF ablation procedures makes this complication noteworthy. Post-PVI, PVS incidence is difficult to estimate because it is often insidious, and the long-term sequelae of mild to moderate PVS remain unclear. Approximately 30%–40% of patients have detectable PV narrowing on cross-sectional imaging at 6 months post-PVI. However, severe PVS is rare, with incidence varying from 0.5% to 4.0% at various timepoints to complete absence in randomized trials (1). When PVS/O occurs, it can increase blood resistance in the affected veins, leading to pulmonary venous hypertension and pulmonary lobe edema (2). Affected alveoli are affected by ischemia and tissue edema, leading to atelectasis, infarction, or infection (3). In the 2012 Heart Rhythm Society/European Heart Rhythm Association/European Cardiac Arrhythmia Society Expert Consensus Statement, PVS is defined as a reduction of a PV diameter and classified as mild (<50%), moderate (50%–70%), and severe (>70% diameter reduction) (4). The clinical manifestations of PVS typically present 3–6 months following RFA for AF. While most patients are asymptomatic, some may experience symptoms such as severe dyspnea, cough, chest pain, and hemoptysis. Complications like recurrent pneumonia, pleural effusion, pulmonary parenchymal consolidation, and pulmonary hypertension can also arise (5). Diagnosis of PVS can be made using computed tomography (CT), magnetic resonance imaging, or transesophageal echocardiography. Treatment for severe cases depends on symptoms and may involve balloon angioplasty, stent placement, or even lobectomy if other interventions fail (2). Stenting is a common treatment method with a low incidence of in-stent restenosis (ISR), particularly around 10% for relatively large stents. When treating PVS/O, PVS stenting has shown to offer better long-term patency compared to balloon angioplasty (2). Larger diameter stents (≥8 mm) may carry a lower risk of restenosis, and reports suggest that pulmonary consolidation can be reversed once clinical symptoms are relieved (5). Therefore, we used a bare metal stent with a diameter of 9 mm and a length of 20 mm during the operation. However, close monitoring of the patient's condition is still necessary. If symptoms related to PV occlusion reappear, a PV CT examination should be promptly conducted. In cases of confirmed in-stent restenosis, balloon angioplasty can be attempted, with thoracoscopic lobectomy being a feasible and safe option if the former is ineffective. Thoracoscopic resection of affected lung lobes has been demonstrated to be feasible and safe in cases of massive hemoptysis caused by complete PV occlusion after RFA for AF (6). Are there better surgical options to avoid PVS/O caused by thermal ablation? In the last years, a nonthermal energy source, pulsed field ablation (PFA), has emerged as an alternative to RF ablation or cryoablation. This technology consists of the delivery of very short-duration, high-amplitude electrical fields that determine myocyte death through electroporation almost in the absence of tissue heating. PFA was shown to be associated with negligible risk of damage to structures surrounding PVs, reduced risk of PV stenosis, short procedure duration, and similar efficacy outcomes compared with thermal ablation. Therefore, PFA is being increasingly performed (7). Researchers have introduced the novel flexible tip TactiFlex™ (TFSE) catheter. An increase in radiofrequency power (up to 50 W) during RFA of AF was recently proposed as a way to reduce procedural duration and increase efficacy, without compromising safety. The high-power, short-duration (HPSD) approach is based on the concept of maximizing resistive heating, while minimizing conductive heating and, therefore, producing wider and shallower ablation lesions, while limiting damages to adjacent extra-cardiac structures like the oesophagus and phrenic nerve (8). It may have more advantages compared with standard-power, long-duration (SPLD). Posteriorwallablation (PWA) iscommonlyaddedto PVI during catheter ablation (CA) of persistent atrial fibrillation. Very-high-power short-duration (vHPSD) PWA plus PVI may be faster and as safe as SP CA among patients with persistent AF, with a trend for superior efficacy (9).

Symptoms began to manifest 7 months post-radiofrequency ablation of AF, with a 3-month delay in reaching a definitive diagnosis. This case highlights the importance of recognizing complications such as PVS/O following the ablation procedure, which can result in diagnostic delays. Treatment was initiated 8 months after symptom onset, with the patient experiencing recurrent hemoptysis necessitating coil closure of the bleeding artery and subsequent stent implantation in the occluded PV. Following the procedures, the patient's symptoms improved significantly, as evidenced by follow-up imaging data. After 22 months post-operation, the patient remains stable with no signs of recurrence. This case serves as a reminder that patients with a history of RFA for AF should consider the possibility of PVS/O if they exhibit pneumonia-related symptoms, particularly if the pneumonia is persistent.

## Data Availability

The original contributions presented in the study are included in the article/Supplementary Material, further inquiries can be directed to the corresponding author/s.
